# Influence of the Mais Médicos (More Doctors) Program on health services access and use in Northeast Brazil

**DOI:** 10.11606/S1518-8787.2019053001571

**Published:** 2019-12-02

**Authors:** Rogério Fabiano Gonçalves, Adriana Falangola Benjamin Bezerra, Oswaldo Y. Tanaka, Carlos Renato dos Santos, Keila Silene de Brito e Silva, Islândia Maria Carvalho de Sousa

**Affiliations:** I Doutorado em Saúde Pública, Instituto Aggeu Magalhães, IAM/Fiocruz-PE. Recife, PE, Brasil.; Universidade de Pernambuco Campus Petrolina, Colegiado de Fisioterapia. Petrolina, PE, Brasil.; II Universidade Federal de Pernambuco, Departamento de Medicina Social, Centro de Ciências da Saúde. Recife, PE, Brasil.; III Universidade de São Paulo, Faculdade de Saúde pública, Departamento de Prática de Saúde Pública. São Paulo, SP, Brasil.; IV V Universidade Federal de Pernambuco, Centro Acadêmico de Vitória, Núcleo de Saúde Coletiva. Vitória de Santo Antão, PE, Brasil.; VI Instituto Aggeu Magalhães, IAM/Fiocruz-PE. Recife, PE, Brasil.

**Keywords:** Distribution of Physicians, Access to Health Services, Health Plans and Programs, Allocation of Resources for Health Care, Primary Health Care

## Abstract

**OBJECTIVE:**

To evaluate the influence of the *Mais Médicos* (More Doctors) Program on the performance of primary health care by quantifying health services access and use in Northeast Brazil, based on the population size of the municipalities, on the financial investment in health, and on the number of physicians in the family health teams.

**METHOD:**

Evaluative research of quantitative nature. Access was evaluated by the population coverage ratio of the Family Health Strategy and use of health services, which were measured by medical appointments conducted between April 2013 and September 2015. We defined processes for database selection, adjustment, and validation, including explanatory variables for a sample of 896 municipalities. The analysis was based on the time periods before and after the implementation of the program. The Wilcoxon signed-rank test and non-parametric alternatives constituted statistical tests in the comparative analysis of the data.

**RESULTS:**

A 19.2% increase was observed in the number of medical appointments between the first six months and the final six months of the data series. In this period, the median appointments in municipalities with up to 5,000 inhabitants increased from 701.0 to 768.0; while in those with more than 100,000 inhabitants it decreased from 285.5 to 280.0 (p < 0.05). Between April 2013 and September 2015, the median coverage ratio of the family health teams increased from 89.2% to 95.3%, approaching 100% in the municipalities with up to 20,000 inhabitants.

**CONCLUSIONS:**

The study highlights the expansion of access and use of primary health care services in the northeast region after the implementation of the *Mais Médicos* (More Doctors) Program. Between April 2013 and September 2015, the coverage of family health teams and the production of medical appointments increased, constituting important achievements for SUS.

## INTRODUCTION

Information on the National Health Survey (NHS) conducted in 2013 indicated inequalities in health services access and use among Brazilian regions, situations also found in previous editions of the survey^1 , 2^. According to NHS, the North and Northeast recorded the lowest ratios of medical appointments in the country^3^. As important challenges for tackling these inequalities, the proposal was to correct the distribution of physicians in the national territory and bring medical care to regions with insufficient professionals in primary health care (PHC)^4^.

In 2013, the Brazilian Ministry of Health (MS) introduced the *Mais Médicos* (More Doctors) Program in the Unified Health System (SUS). With the PMM, an emergency physician allocation process was initiated, and more than 4,000 municipalities began to have the aid of the MS for the allocation of these professionals. In the first two years, more than 18,000 physicians were integrated into the PHC workforce, with the Northeast being one of the most favored regions^5 , 6^.

Considering the problem of lacking medical care in PHC, the considerable contribution of physicians assured by PMM, and the significant number of municipalities participating in the program, the evaluation of results is strategic for planning and managing PHC. In addition, a restructuring of the PMM was initiated in 2018, with prospect of its substitution or closure, which makes it fundamental to verify how the program influenced health services access and use, in different contexts, to highlight its potential and its limits.

Thus, this study evaluates the influence of PMM on the performance of PHC by quantifying health services access and use in the northeast region, based on the population size of the municipalities, the financial investment in health, and the number of physicians in the family health teams.

## METHODS

Evaluative research was carried out by obtaining secondary quantitative data. Access, “as a dimension of health systems performance associated with supply,”^7^ was evaluated by the estimated population coverage ratio of the family health teams (FHt). We used FHt coverage data from April 2013 to September 2015, obtained from the Primary Care Department website.

The use of health services was evaluated by the medical appointments conducted in the Family Health Strategy (FHS) and registered in the SUS Outpatient Information System (SIA/SUS). The data reference period was also delimited from April 2013 to September 2015, totaling 30 months. It included the first six months of the medical appointment series (April to September 2013) as a period not influenced by the physicians’ work in PMM and the following twenty-four months (October 2013 to September 2015) as the starting point of the PMM results for the evaluated data. The months of August and September 2013 were not included in the program’s results period, since they were months of convocation, integration, and adaptation for the new physicians.

The important data absence of the FHS medical appointments in SIA/SUS precluded extending the evaluation to the months prior to April 2013. The series’ last month, September 2015, was defined considering the program’s two-year validity period, based on the established time frame.

In addition to the variables above, the analysis included: the number of PMM physicians up to the end of the fifth cycle of adherence of professionals, in June 2014 (data obtained from the PMM website and from records of the program provided by the Ministry of Health); the number of physicians working in the FHt in April 2013 and September 2015, respectively the initial and final months of the medical appointments period determined for this analysis, obtained from the DATASUS website; the total per capita spending in health in 2013 and 2015 and over the period, obtained from the Health Public Budgets Information System website; and the mean population size of the municipalities, calculated for the 2013–2015 period, according to data from the population estimates informed in DATASUS.

As a development of the analysis of FHS appointments, we calculated the daily production of appointments by professional (appointments/day). We established the calculation based on the ratio between the total FHS appointments and the working days of the respective period. This result was divided by the mean number of physicians working in the FHS between April 2013 and September 2015.

**Figure 1:**
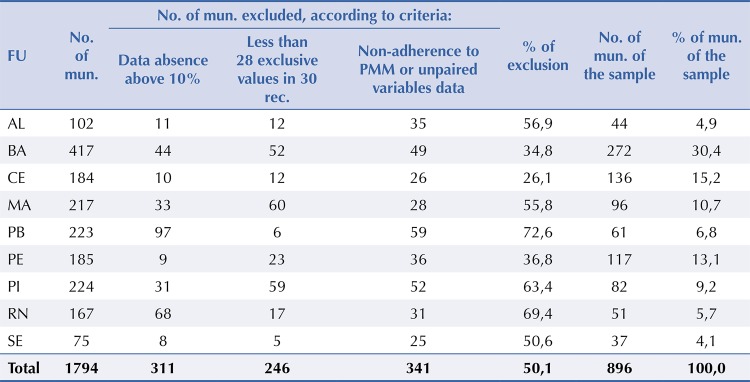
Characterization of the sample of municipalities in the research, according to exclusions conducted and distribution by federation units of the northeast region. FU: Federation Units; mun.: municipalities; rec.: records

Source: prepared by the authors based on the research data.

The sample of Northeast (NE) municipalities was determined by applying exclusion criteria, resulting in 896 municipalities (49.9% of the NE total and 68.7% of those with PMM), as shown in Figure 1 . We excluded municipalities with more than 10% absence of data of FHS appointments in the 30-month series, with less than 28 exclusive values (number of appointments in each month, different from zero, which were not repeated), and those that did not adhere to the PMM or did not present information for the pairing of analysis variables. These criteria aimed to provide quality and enhance the validation of the database.

Data were prepared by detecting and converting absent and discrepant values, and the color saturation scale was used in Excel (Microsoft Office) as a conditional formatting criterion. In this procedure, we ranked the sum of appointments per municipality, which were grouped according to the similarity of scores. In the groups, we observed the tonality variation of the green, yellow, and red colors, aiming to trace the most intense tones.

Using the color scales, 26,880 values were checked, and those traced at the extremes of variation were converted (intense green and intense red, respectively values far above the others and far below or absent). The yellow color, intermediate range of the color variation, indicated a regularity pattern of the values in the data series, and it was not modified. For conversion, we adopted as a rule the mean of four values in the data series adjacent to the value to be converted. Thus, the value to be converted could be to the left, to the right, or between the four surrounding values that determined the mean.

After the conversions, a more uniform pattern of colors was assumed by the distribution of data, showing a reduction in discrepancies. We highlight the example of a municipality in Bahia, which initially recorded a monthly mean of 23,270 appointments in the data series – a higher number than that of the capital city, Salvador. When a discrepant value equal to 509,958 was detected among those registered, its conversion reduced that municipality’s mean to 6,485 appointments, a more realistic value in relation to the data series.

Data analysis was initiated by the Kolmogorov-Smirnov (K-S) adherence test between two samples, which aimed to determine the impact of modifications in the database. The appointments information density was compared between the original and the modified database.

The free software R was used as support for statistical analysis. For each variable of interest, the normality of data distributions was checked by the Shapiro-Wilk test, which indicated non-normal distributions. Accordingly, the Wilcoxon signed-rank test was defined as a non-parametric alternative of analysis, establishing a significance of 5%. In some circumstances, we applied the Kruskal-Wallis test with Dunn’s post-test or the chi-square test, according to the purpose.

As for the ethical aspects, considering that this investigation uses data with public access via the Internet, available in official information systems, it required no submission for assessment by ethics committee.

## RESULTS

The adherence analysis by the K-S test, comparing the original database with the modified database, indicated that the data remained unchanged in 699 municipalities of the sample (78.0%), small changes were detected in 184 (20.5%), and the changes were moderate in 13 (1.5%). These results confirmed that the samples originated from the same distribution, preserving the correspondence between them.

### FHt coverage

A significant increase was found in FHt coverage with the PMM. This advance is evidenced in the comparison between April 2013 and September 2015. In the aforementioned interval, the median increased from 89.2% to 95.3% (p < 0.05, Wilcoxon test).

**Figure 2:**
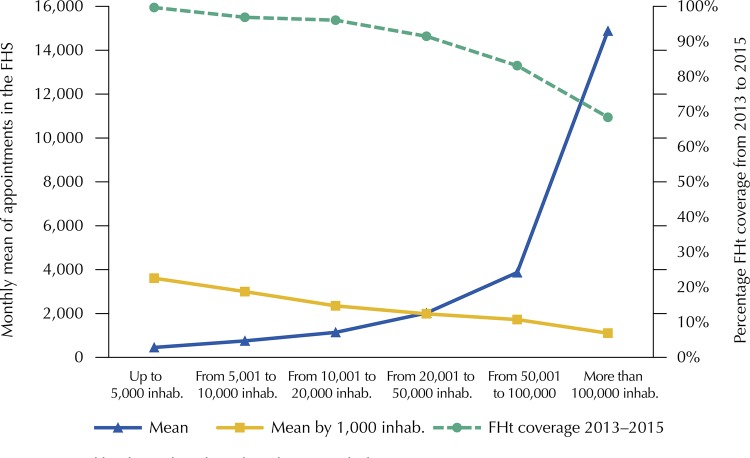
Mean and mean adjusted for 1,000 inhabitants of monthly appointments in the Family Health Strategy in a sample of municipalities participating in the *Mais Médicos* (More Doctors) Program in the northeast region, according to population size and percentage family health teams coverage, April 2013 to September 2015. FHS: Family Health Strategy; FHt: Family Health teams.

Source: prepared by the authors based on the research data.

In municipalities with up to 20,000 inhabitants, coverage approached 100% at the end of the period under analysis. In municipalities in the range of 50,001 to 100,000 inhab., coverage increased from 75.9% to 90.3% in the period; in those with more than 100,000 inhab., coverage increased from 64.8% to 72.1% (p < 0.05, Wilcoxon test). Figure 2 shows the differences in the FHt coverage ratio between population sizes.

### FHS appointments

**Figure 3:**
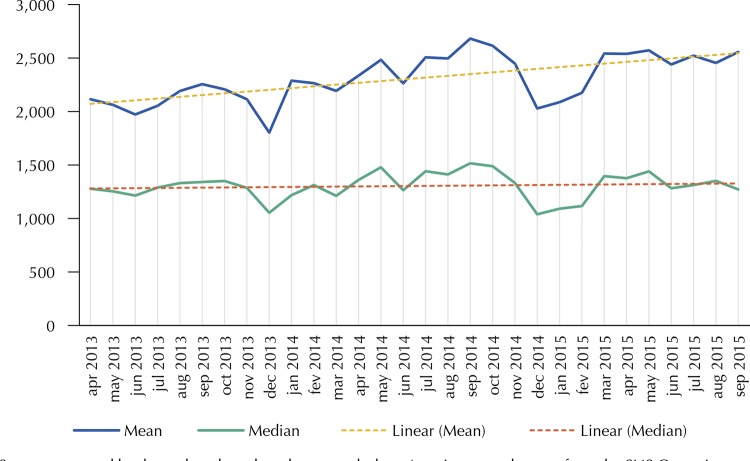
Mean and median monthly appointments in the Family Health Strategy in a sample of municipalities participating in the *Mais Médicos* (More Doctors) Program in Northeast Brazil, April 2013 to September 2015.

Source: prepared by the authors based on the research data. Appointments data are from the SUS Outpatient Information System (SIA/SUS), adjusted according to the procedures described in the Methods section.

There was oscillation in the monthly production of appointments in the analyzed sample. We observed, in most of the reference period, decreased appointments in June and December, with peaks in September, especially when considering the behavior of the means in 2013 and 2014 ( Figure 3 ).

The comparison of the number of appointments in the first six months with the final six months of the data series showed a 19.2% increase in the production, confirmed by the Wilcoxon signed-rank test (p < 0.05). In these six-month periods, the means ranged from 2,108.7 in 2013 to 2,514.2 in 2015; while the medians ranged, respectively, from 2,093.8 to 2,507.9.

Analysis of mean appointments by population size – when adjusted by 1,000 inhab. – shows that, proportionally to population, smaller municipalities had more FHS appointments than larger ones and presented higher FHt coverage ( Figure 2 ).

Median of appointments in municipalities with up to 5,000 inhabitants increased from 701.0 to 768.0 – a 9.6% increase between the first six months and the final six months of the data series. In those with more than 100,000 inhabitants, it decreased from 285.5 to 280.0 – a reduction of 1.8%. The highest variation between the sizes was that of municipalities with 50,001 to 100,000 inhabitants, with an increase of 18.2%; however, with lower medians than municipalities with up to 5,000 inhabitants (p < 0.05, Wilcoxon test).

### Appointments/day by physician in the FHS

Appointments/day by professional decreased between the initial and the final six-month period of the data series. The median in the first six-month period was 10.1, and decreased to 9.4 (p < 0.05, Wilcoxon test). Despite the decreased daily production of appointments by professional, general appointments increased, which suggests influence of the increased provision of medical care by the PMM.

Municipalities with up to 5,000 inhabitants presented a higher number of appointments/day by professional during the PMM, with median equal to 11.3, while the median of municipalities with more than 100,000 inhabitants was 9.8. Dunn’s post-test indicated that the production of appointments in municipalities with up to 5,000 inhabitants also differed from that of municipalities of other sizes (p < 0.05).

### Physicians in the FHt

Although the overall number of PMM physicians is lower in municipalities with up to 10,000 inhabitants, the ratio of physicians of the program in relation to the total physicians in the FHt was higher in these locations ( Table 1 ). In municipalities with up to 5,000 inhabitants, this ratio was 61.4%; while in those with 5,001 to 10,000 inhabitants, it was 46.8%; in municipalities with more than 100,000 inhabitants, it decreases to 24.2%.

**Table 1:** Quantitative distribution of physicians in the Family Health strategy teams in September 2015 and indicators derived from a sample of municipalities participating in the *Mais Médicos* (More Doctors) Program in the Northeast region, according to population size.

	Population size of the municipalities					
	Up to 5,000 inhab.	5,001 to 10,000 inhab.	10,001 to 20,000 inhab.	20,001 to 50,000 inhab.	50,001 to 100,000 inhab.	More than 100,000 inhab.
FHt total physicians	114	442	1,776	2,759	1,927	3,322
PMM total physicians	70	207	728	1,139	780	805
FHt physicians/ 10,000 inhab.	5.0	4.8	4.0	3.4	2.9	1.8
FHt phys./10,000 without PMM	1.9	2.5	2.4	2.0	1.7	1.4
PMM/FHt physicians ratio	61.4%	46.8%	41.2%	41.3%	40.5%	24.2%
sample mun. %, n = 896	6.8%	13.7%	33.4%	30.0%	10.9%	5.1%

Source: prepared by the authors based on the research data.

FHt: Family Health teams; PMM: *Mais Médicos* (More Doctors) Program; phys.: physicians; PMM/FHt physicians ratio: ratio of PMM physicians in relation to the physicians of the FHt in which the program physicians are allocated; mun.: municipalities

In September 2015, the ratio of physicians per 10,000 inhabitants in the FHt in municipalities with smaller population was more than double in relation to that of municipalities with more than 100,000 inhabitants, respectively 5.0 and 1.8 ( Table 1 ). When physicians allocated by the PMM in the FHt are not accounted, this mean would decrease from 5.0 to 1.9 in municipalities with up to 5,000 inhab. and from 1.8 to 1.4 in those with more than 100,000 inhab. Although these figures constitute a simulation based on the non-implementation of the PMM or on the hypothetical number of physicians in the FHt without the program, the data denote its importance in smaller municipalities.

In April 2013, there were 8,982 physicians in the FHt of the municipalities in the sample and, in September 2015, there were 10,308. The observed increase was 1,326 professionals. However, in June 2014, the PMM had integrated 3,729 physicians into these teams, a number that supposedly increased up to September 2015. Therefore, there is a difference of at least 2,403 professionals (64.4% of the physicians integrated by the PMM). This number would raise the number of physicians in September 2015 to 12,711.

### Total Per Capita Spending in Health

**Figure 4:**
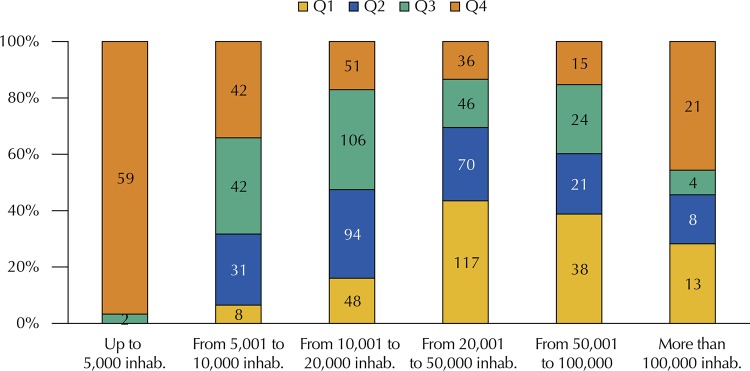
Number of municipalities by mean population size in 2013–2015, according to quartiles of the mean total per capita spending in health for the period, in a 100% stacked column chart. Note: each quartile represents a set of 224 municipalities (25% of the sample), in ascending order, with the first quartile (Q1) including municipalities with lower per capita spending and the fourth quartile (Q4) including municipalities with higher per capita spending.

Source: prepared by the authors based on the research data.

The investment in health, traced by the analysis of total per capita spending in health, shows that municipalities with up to 10,000 inhab., especially those with up to 5,000 inhab., had higher spending in health per inhabitant compared with municipalities with other population sizes. Figure 4 illustrates this reality, with significant association between the variables by the chi-square test (p < 0.05).

The median of total spending in the sample increased from R$ 386.0 in 2013 to R$ 447.7 in 2015 (p < 0.05, Wilcoxon test). In both years, it was higher in municipalities with up to 5,000 inhab.: R$ 566.1 in 2013 and R$ 661.4 in 2015. We also observed that increasing levels of production of appointments by the FHt were associated with higher levels of per capita investment in health from 2013 to 2015 (p < 0.05, chi-square).

## DISCUSSION

The adjustments made in the preparation of the database proved to be adequate, contributing to the recovery of sensitive data, which favored the analysis. Using the Excel color saturation scale enabled identifying inconsistencies in the data reported by the SIA/SUS, interpreted as errors or weaknesses in obtaining, feeding or recording data processes.

The reduction in the peak of appointments in the PHC in cyclical months, observed in the results, is a subject poorly explored in the literature, limiting a comparative approach. The reductions are related to the traditional festivities (involving the *São João* celebration in the Northeast, Christmas, and New Year’s Eve), close to periods of work and school holidays, in addition to the period of semiannual and annual consolidation of care production in SUS.

The increase in appointments in the PHC, according to data presented, corroborates the result of a multicenter study conducted by the PMM Observatory Network, which analyzed data from 3,000 municipalities in the country^8^. Between 2013 and 2015, there was a 33% increase in the number of appointments in the municipalities participating in the program, over 15% in the non-participating municipalities. In this study, the increase in the participating municipalities was lower: 19.2%. As the data from the Observatory network were nationwide and disseminated in a grouped manner, specific comparisons were impossible.

We observed better performance of municipalities with smaller population size in the production of appointments of the FHS. This performance should reflect not only the greater coverage of the strategy in these municipalities, but also the configuration of health care. It is understood that, in smaller municipalities, the PHC tends to be the main or single local public health network configuration, centered on the provision of medical care by the FHS, justifying greater demand for services in this context. The importance of the PMM in providing access to municipalities with greater difficulties in obtaining professionals is also considered. In larger municipalities, the greater supply of physicians outside the FHS, especially of clinicians and pediatricians, expands the options of entry and access to SUS^9^.

As for the daily production of appointments by professionals, two assumptions related to the influence of PMM can clarify the observed reduction: the greater regularity of the presence of physicians in the services and the reduction of turnover of professionals. These two factors tend to strengthen the professional-user bond, reduce the population’s insecurity regarding the absence of physicians, minimize queues and waiting time, order the care schedule, and avoid repressed demands^10^. Thus, by increasing the population’s confidence in the regular provision of medical care in the FHS, the demand, programmed or spontaneous, would be redistributed into weekdays and would lead to decreased daily appointments. A qualitative study conducted in two *Quilombola* communities reported that the PMM caused changes. The work highlighted the increase in the frequency of physicians in the services and in the home visits, which impacted the optimization of appointment schedules, facilitating the use of these services by the population^10^.

In addition to these factors, we also emphasize as assumptions the extension of the duration of appointments and the improvement in the quality of care. The supervised monitoring of physicians of the program and the introduction of exchange professionals, with training and experience working in the PHC in other contexts, are elements that could influence changes in the medical performance. This is not just an increase in the number of physicians, but also the implementation of the process of continuing education and follow-up. These elements need further deepening with qualitative studies, which may improve the program.

In a qualitative research conducted with FHS users in Recife (PE), the quality of care provided by Cuban physicians was emphasized by the conduct of differentiated clinical practices. According to the interpretation of the reports, the care provided by them was characterized by “commitment, quality listening, comprehensive view of the subject, and consideration as to the social determinants of the health-disease process.”^11^

As for the expansion of the medical workforce in the PHC with the PMM, the results of this study are corroborated by the literature. Analysis of medical care provision in a sample of 3,755 municipalities in all regions of the country concluded that there was greater impact of the PMM on municipalities with up to 10,000 inhab., with a 22.2% increase in the medical workforce^9^. The significant increase in the provision of health care in the FHt in these municipalities was justified by the greater demand since it is predominantly organized by the PHC.

It is known that in municipalities with larger population, due to the greater socioeconomic development and concentration of services of medium and high complexities, there are more attractive aspects for the fixation of physicians^12^. Accordingly, these municipalities depend less on provision policies, a fact that justifies the greater ratio of PMM physicians in the FHt of municipalities with smaller population size^13^. As for the real increase in the number of physicians in the FHt being lower than expected, other studies also reported this situation, justifying the replacement of professionals or the insertion of physicians in incomplete teams^9 , 13 , 14 , 15^.

The increased FHt coverage in municipalities of all population sizes, approaching 100% in those with up to 20,000 inhabitants, shows the importance of PMM as to meeting the demand for medical care in PHC, having a direct influence on the increase observed in appointments. This result is consistent with the data presented in a nationwide study that showed a 16.2% increase in FHt coverage from 2012 to 2015^15^. The highest coverage rates were observed in municipalities with less than 30,000 inhab., with mean of 98.4% in 2015; in municipalities with 100,000 to 200,000 inhab., the mean corresponded to 48.9%.

Regarding the analysis of investment in health, we highlight the higher per capita investment of municipalities with smaller population. Although the PMM enables relieving the municipalities in relation to spending with the provision of physicians^16^, the increase in these municipalities tends to reflect the greater production of appointments in the FHS and the expansion of FHt coverage.

As this research is consolidated with secondary data and records of appointments on a regional scale, these aspects are recognized as limitations of the method in this study. Thus, we recommend studies that can explore the local context of PHC and the possible impacts on actions and services considering the current guidelines.

## CONCLUSIONS

The results of this study show evidence that there was increase in the production of appointments in PHC in the Northeast, characterizing the expansion of access to and use of services in this context with the implementation of PMM. Between April 2013 and September 2015, there was an effective increase in FHt coverage, constituting an important achievement for SUS.

The better performance in the production of appointments obtained by municipalities with smaller population size, because of higher per capita investments in health and the centrality of PHC in the provision of actions and services, suggests the need to advance in the discussion of equitable allocation of resources in health and to strengthen the care network, with emphasis on regionalization.

The findings may contribute to the recent discussions about the effects of PMM, as they evidence the influence of the program in different contexts. The future alternatives to the program to be implemented may, based on the advances and difficulties of the PMM, invest in strategies that increase the positive results achieved and minimize the limits, in order to continue the population’s access in the PHC.
