# Supplementary material for: Influence of the Mais Médicos (More Doctors) Program on health services access and use in Northeast Brazil
Source: Rev Saude Publica. 2019 Dec 2;53:110. doi: 10.11606/S1518-8787.2019053001571 (PMC6904122; doi:10.11606/S1518-8787.2019053001571)
Supplement: Translation

# Translation: Influência do Mais Médicos no acesso e na utilização de serviços de saúde no Nordeste

**Authors:** Rogério Fabiano Gonçalves, Adriana Falangola Benjamin Bezerra, Oswaldo Y. Tanaka, Carlos Renato dos Santos, Keila Silene de Brito e Silva, Islândia Maria Carvalho de Sousa

**Affiliations:** I Doutorado em Saúde Pública, Instituto Aggeu Magalhães, IAM/Fiocruz-PE. Recife, PE, Brasil.; Universidade de Pernambuco Campus Petrolina, Colegiado de Fisioterapia. Petrolina, PE, Brasil; II Universidade Federal de Pernambuco, Departamento de Medicina Social, Centro de Ciências da Saúde. Recife, PE, Brasil; III Universidade de São Paulo, Faculdade de Saúde pública, Departamento de Prática de Saúde Pública. São Paulo, SP, Brasil; IV V Universidade Federal de Pernambuco, Centro Acadêmico de Vitória, Núcleo de Saúde Coletiva. Vitória de Santo Antão, PE, Brasil; VI Instituto Aggeu Magalhães, IAM/Fiocruz-PE. Recife, PE, Brasil

**Keywords:** Distribuição de Médicos, Acesso aos Serviços de Saúde, Planos e Programas de Saúde, Alocação de Recursos para a Atenção à Saúde, Atenção Primária à Saúde

## Abstract

**OBJETIVO:**

Avaliar a influência do programa Mais Médicos no desempenho da atenção primária à saúde pelo dimensionamento do acesso e da utilização de serviços de saúde na região Nordeste, baseado no porte populacional dos municípios, no investimento financeiro em saúde e no quantitativo de médicos nas equipes de saúde da família.

**MÉTODO:**

Pesquisa avaliativa de natureza quantitativa. O acesso foi avaliado pela proporção de cobertura populacional da Estratégia Saúde da Família e a utilização de serviços de saúde, aferida pelas consultas médicas realizadas nesse âmbito entre abril de 2013 e setembro de 2015. Foram definidos processos de seleção, ajuste e validação do banco de dados, com inclusão de variáveis explicativas, que resultaram em amostra de 896 municípios. A análise fundamentou-se em recorte temporal anterior e posterior à implantação do programa. O teste de Wilcoxon pareado e alternativas não paramétricas constituíram provas estatísticas na análise comparativa dos dados.

**RESULTADOS:**

Houve aumento de 19,2% no quantitativo de consultas médicas entre os seis meses iniciais da série de dados e os seis meses finais. Nesse período, a mediana de consultas nos municípios com até 5.000 habitantes aumentou de 701,0 para 768,0; já naqueles com mais de 100.000 habitantes, reduziu de 285,5 para 280,0 (p < 0,05). Entre os meses de abril de 2013 e setembro de 2015, a mediana da proporção de cobertura de equipes de saúde da família passou de 89,2% para 95,3%, aproximando-se de 100% nos municípios com até 20.000 habitantes.

**CONCLUSÕES:**

O estudo evidencia a ampliação do acesso e da utilização de serviços na atenção primária à saúde na região Nordeste a partir da implantação do programa Mais Médicos. Entre abril de 2013 e setembro de 2015, a cobertura de equipes de saúde da família e a produção de consultas médicas cresceram, constituindo importantes conquistas para o SUS.

## INTRODUÇÃO

Informações da Pesquisa Nacional de Saúde (PNS) realizada em 2013 indicaram desigualdades no acesso e na utilização de serviços de saúde entre as regiões do Brasil, situações também identificadas em edições anteriores da pesquisa^1 , 2^. Conforme a PNS, o Norte e o Nordeste registravam as menores proporções de consultas médicas do país^3^. Como importantes desafios para o enfrentamento dessas desigualdades, propunham-se corrigir a distribuição de médicos no território nacional e levar assistência médica para regiões com insuficiência de profissionais na atenção primária à saúde (APS)^4^.

Em 2013, o Ministério da Saúde (MS) implantou o programa Mais Médicos (PMM) no Sistema Único de Saúde (SUS). A partir do PMM, um processo emergencial de alocação de médicos foi iniciado e mais de 4.000 municípios passaram a ter o auxílio do MS para a alocação desses profissionais. Nos primeiros dois anos, foram integrados mais de 18.000 médicos na força de trabalho da APS, sendo o Nordeste uma das regiões mais favorecidas^5 , 6^.

Diante do problema da carência de assistência médica na APS, do considerável aporte de médicos assegurado pelo PMM e do expressivo quantitativo de municípios participantes do programa, a avaliação dos seus resultados é estratégica para o planejamento e a gestão da APS. Além disso, em 2018, iniciou-se uma reestruturação do PMM, com perspectivas de sua substituição ou seu encerramento, o que torna basilar a constatação de como o programa influenciou o acesso e a utilização de serviços de saúde, em diferentes contextos, para evidenciar seu potencial e seus limites.

Assim, este estudo avalia a influência do PMM no desempenho da APS pelo dimensionamento do acesso e da utilização de serviços de saúde na região Nordeste, a partir do porte populacional dos municípios, do investimento financeiro em saúde e do quantitativo de médicos nas equipes de saúde da família.

## MÉTODOS

Foi realizada pesquisa avaliativa por meio da obtenção de dados quantitativos secundários. O acesso, “enquanto dimensão do desempenho dos sistemas de saúde associada à oferta”^7^, foi avaliado pela proporção da cobertura populacional estimada das equipes de saúde da família (eSF). Foram utilizados dados de cobertura das eSF dos meses de abril de 2013 e setembro de 2015, obtidos no site do Departamento de Atenção Básica.

A utilização de serviços de saúde foi avaliada pelas consultas médicas realizadas na Estratégia Saúde da Família (ESF) e registradas no Sistema de Informações Ambulatoriais do SUS (SIA/SUS). O período de referência dos dados também foi delimitado de abril de 2013 a setembro de 2015, totalizando 30 meses. Ele incluiu os seis meses iniciais da série de consultas médicas (abril a setembro de 2013) como período não influenciado pelo trabalho dos médicos do PMM e os vinte e quatro meses seguintes (outubro de 2013 a setembro de 2015) como o ponto de partida dos resultados do PMM para os dados avaliados. Os meses de agosto e setembro de 2013 não foram incluídos no período de resultados do programa, visto que foram meses de convocação, integração e adaptação dos médicos recém-chegados.

A importante ausência de dados das consultas médicas da ESF no SIA/SUS não permitiu estender a avaliação para os meses anteriores a abril de 2013. A definição do último mês da série, setembro de 2015, levou em consideração o período de dois anos de vigência do programa, a partir do recorte temporal estabelecido.

Além das variáveis citadas, foram incluídos na análise: o quantitativo de médicos do PMM até o final do quinto ciclo de adesão de profissionais, em junho de 2014 (dados obtidos no site do PMM e por meio de registros do programa cedidos pelo Ministério da Saúde); o quantitativo de médicos que atuavam nas eSF nos meses de abril de 2013 e setembro de 2015, respectivamente os meses de início e término do período de consultas médicas determinados para esta análise, obtidos no site do DATASUS; a despesa total em saúde per capita nos anos de 2013 e 2015 e em todo o período, obtidas no site do Sistema de Informações sobre Orçamentos Públicos em Saúde; e a média do porte populacional dos municípios, calculada para o período de 2013 a 2015, conforme dados das estimativas populacionais informadas no DATASUS.

Como desdobramento da análise das consultas médicas realizadas na ESF, foi calculada a produção diária de consultas por profissional (consultas/dia). O cálculo foi estabelecido a partir da razão entre o total de consultas médicas da ESF e os dias úteis do respectivo período. Esse resultado foi divido pela média da quantidade de médicos atuantes na ESF entre os meses de abril de 2013 e setembro de 2015.

A amostra de municípios do Nordeste (NE) foi determinada pela aplicação de critérios de exclusão, resultando em 896 municípios (49,9% do total do NE e 68,7% daqueles com PMM), como apresentado na Figura 1 . Foram excluídos os municípios com mais de 10% de ausência de dados das consultas médicas da ESF na série de 30 meses, com menos de 28 valores exclusivos (número de consultas em cada mês, diferentes de zero, que não se repetem) e aqueles que não fizeram adesão ao PMM ou não apresentavam informações para o pareamento de variáveis da análise. O intuito desses critérios foi qualificar e potencializar a validação do banco de dados.

**Figura 1:** Conformação da amostra de municípios da pesquisa, segundo exclusões realizadas e distribuição por unidades federativas da região Nordeste. UF: Unidades da Federação; mun.: municípios; reg.: registros

Fonte: elaboração própria com base nos dados da pesquisa.

Procedeu-se à preparação dos dados pela detecção e conversão de valores ausentes e discrepantes, sendo utilizada escala de saturação de cores no Excel (Microsoft Office) como critério de formatação condicional. Nesse procedimento, foi estabelecido um *ranking* da soma de consultas médicas por município, agrupados conforme semelhança de pontuação. Nos grupos, foi observada a variação de tonalidade das cores verde, amarelo e vermelho, buscando-se identificar os tons mais intensos.

A partir das escalas de cor, 26.880 valores foram submetidos à verificação, e aqueles identificados nos extremos de variação foram convertidos (verde intenso e vermelho intenso, respectivamente valores muito acima dos demais e muito abaixo ou ausentes). A cor amarela, faixa intermediária da variação de cores, sinalizava um padrão de regularidade dos valores na série de dados, não sendo objeto de modificação. Para a conversão, foi adotada como regra a média de quatro valores na série de dados adjacentes ao valor a ser convertido. Dessa forma, o valor a ser convertido poderia estar à esquerda, à direita ou entre os quatro valores circundantes que determinavam a média.

Após as conversões, um padrão mais uniforme de cores era assumido pela distribuição de dados, demonstrando redução das discrepâncias. Cita-se o exemplo de um município da Bahia que, inicialmente, registrava média mensal de 23.270 consultas médicas na série de dados – número superior ao da capital, Salvador. Ao ser detectado valor discrepante igual a 509.958 entre os registrados, sua conversão reduziu a média desse município para 6.485 consultas, valor mais realista em relação à série de dados.

A análise dos dados foi iniciada pelo teste de aderência de Kolmogorov-Smirnov (K-S) entre duas amostras, que objetivou verificar o impacto das modificações realizadas no banco de dados. Comparou-se a densidade de informações das consultas médicas entre o banco de dados original e o modificado.

O software livre R foi utilizado como suporte à análise estatística. Para cada variável de interesse, conferiu-se a normalidade das distribuições de dados pelo teste de Shapiro-Wilk, que indicou distribuições não normais. Nesse sentido, o teste de Wilcoxon pareado foi definido como alternativa não paramétrica de análise, estabelecendo-se significância de 5%. Em algumas circunstâncias foi aplicado o teste de Kruskal-Wallis com pós-teste de Dunn ou o teste do qui-quadrado, conforme a finalidade.

No que se refere aos aspectos éticos da pesquisa, considerando que esta investigação utiliza dados de acesso público pela internet, disponíveis em sistemas de informação oficiais, não se fez necessária submissão para apreciação por comitê de ética.

## RESULTADOS

A análise de aderência pelo teste K-S, comparando o banco de dados original com o modificado, indicou que os dados permaneceram inalterados em 699 municípios da amostra (78,0%), detectaram-se pequenas alterações em 184 (20,5%), e as mudanças foram moderadas em 13 (1,5%). Tais resultados confirmaram que as amostras provinham da mesma distribuição, preservando-se a correspondência entre elas.

### Cobertura das eSF

Houve aumento significativo de cobertura das eSF a partir do PMM. Esse avanço é evidenciado no comparativo entre abril de 2013 e setembro de 2015. A mediana passou, no referido intervalo, de 89,2% para 95,3% (p < 0,05, Wilcoxon pareado).

Nos municípios com até 20.000 hab, a cobertura se aproximou de 100% ao final do período analisado. Nos municípios na faixa de 50.001 a 100.000 hab., a cobertura passou de 75,9% para 90,3% no período; naqueles com mais de 100.000 hab., passou de 64,8% para 72,1% (p < 0,05, Wilcoxon pareado). A Figura 2 ilustra as diferenças na proporção de cobertura de eSF entre os portes populacionais.

**Figura 2:** Média e média ajustada para 1.000 habitantes de consultas médicas mensais na Estratégia Saúde da Família em amostra de municípios participantes do programa Mais Médicos na região Nordeste, segundo porte populacional e percentual de cobertura de equipes de saúde da família, abril de 2013 a setembro de 2015. ESF: Estratégia Saúde da Família; eSF: equipes de saúde da família.

Fonte: elaboração própria com base nos dados da pesquisa.

### Consultas Médicas na ESF

Houve oscilação da produção de consultas médicas mensais na amostra analisada. Observou-se, na maior parte do período de referência, declínio das consultas nos meses de junho e dezembro, com ápices em setembro, principalmente ao se considerar o comportamento das médias nos anos de 2013 e 2014 ( Figura 3 ).

**Figura 3:** Média e mediana de consultas médicas mensais na Estratégia Saúde da Família em amostra de municípios participantes do programa Mais Médicos na região Nordeste, abril de 2013 a setembro de 2015.

Fonte: elaboração própria com base nos dados da pesquisa. Os dados de consultas médicas são provenientes do Sistema de Informações Ambulatoriais do SUS (SIA/SUS), ajustados conforme procedimentos descritos na seção Métodos.

A comparação do quantitativo de consultas realizadas nos seis meses iniciais da série de dados com os seis meses finais indicou aumento de 19,2% na produção, confirmado pelo teste de Wilcoxon pareado (p < 0,05). A variação das médias nesse recorte semestral foi de 2.108,7 em 2013 para 2.514,2 em 2015; para as medianas a variação correspondeu, respectivamente, a 2.093,8 e 2.507,9.

A visualização das médias de consultas médicas por porte populacional, quando ajustadas por 1.000 hab., mostra que os municípios de menor porte realizaram, proporcionalmente à população, mais consultas médicas na ESF que os de maior porte e apresentam cobertura de eSF mais elevada ( Figura 2 ).

A mediana de consultas médicas nos municípios com até 5.000 hab. passou de 701,0 para 768,0 – aumento de 9,6% entre os seis meses iniciais da série de dados e os seis meses finais. Naqueles com mais de 100.000 hab., passou de 285,5 para 280,0 – redução de 1,8%. A maior variação entre os portes foi dos municípios de 50.001 até 100.000 hab., com um aumento de 18,2%, todavia com medianas mais baixas que os municípios com até 5.000 hab. (p < 0,05, Wilcoxon pareado).

### Consultas/Dia por Médico na ESF

Observou-se redução de consultas/dia por profissional entre o semestre inicial da série de dados e o final. A mediana no primeiro semestre foi de 10,1, reduzida para 9,4 (p < 0,05, Wilcoxon pareado). Apesar da diminuição na produção diária de consultas por profissional, houve aumento de consultas no geral, o que sugere influência da ampliação da oferta de assistência médica pelo PMM.

Os municípios com até 5.000 hab. apresentaram maior quantitativo de consultas/dia por profissional na vigência do PMM, com mediana igual a 11,3, enquanto a de municípios com mais de 100.000 hab. foi 9,8. O pós-teste de Dunn indicou que a produção de consultas nos municípios de até 5.000 hab. também diferiu daquela dos municípios de outros portes (p < 0,05).

### Médicos nas eSF

Embora o quantitativo geral de médicos do PMM seja menor em municípios de até 10.000 hab., a proporção de médicos do programa em relação à totalidade de médicos nas eSF foi maior nesses locais ( Tabela 1 ). Nos municípios de até 5.000 hab., essa proporção foi de 61,4%; já naqueles de 5.001 a 10.000 hab., foi de 46,8%; em municípios com mais de 100.000 hab., ela cai para 24,2%.

**Tabela 1:** Distribuição quantitativa de médicos nas equipes da Estratégia Saúde da Família em setembro de 2015 e indicadores derivados em amostra de municípios participantes do programa Mais Médicos na região Nordeste, segundo porte populacional.

	Porte populacional dos municípios					
	Até 5.000 hab.	5.001 a 10.000 hab.	10.001 a 20.000 hab.	20.001 a 50.000 hab.	50.001 a 100.000 hab.	Mais de 100.000 hab.
Total de médicos nas eSF	114	442	1.776	2.759	1.927	3.322
Total de médicos do PMM	70	207	728	1.139	780	805
Médicos eSF/ 10.000 hab.	5,0	4,8	4,0	3,4	2,9	1,8
Méd. eSF/10.000 sem PMM	1,9	2,5	2,4	2,0	1,7	1,4
Prop. médicos PMM/eSF	61,4%	46,8%	41,2%	41,3%	40,5%	24,2%
% mun. amostra, n = 896	6,8%	13,7%	33,4%	30,0%	10,9%	5,1%

Fonte: elaboração própria com base nos dados da pesquisa.

eSF: equipes de saúde da família; PMM: programa Mais Médicos; Méd.: médicos; Prop. médicos PMM/eSF: proporção de médicos do PMM em relação aos médicos das eSF nas quais os médicos do programa são alocados; mun.: municípios.

Em setembro de 2015, a razão de médicos por 10.000 hab. nas eSF em municípios de menor porte populacional era mais que o dobro em relação àquela dos municípios com mais de 100.000 hab., respectivamente 5,0 e 1,8 ( Tabela 1 ). Não contabilizando os médicos alocados pelo PMM nas eSF, a referida média passaria de 5,0 para 1,9 nos municípios de até 5.000 hab. e de 1,8 para 1,4 naqueles com mais de 100.000 hab. Embora esses números constituam uma simulação baseada na não implementação do PMM ou no quantitativo hipotético de médicos nas eSF sem o programa, os dados denotam a importância dele nos municípios de menor porte.

Em abril de 2013, havia 8.982 médicos nas eSF dos municípios da amostra e, em setembro de 2015, 10.308. O aumento observado foi de 1.326 profissionais. Todavia, em junho de 2014, o PMM havia integrado 3.729 médicos nessas equipes, número que supostamente cresceu até setembro de 2015. Portanto, existe uma diferença de no mínimo 2.403 profissionais (64,4% dos médicos integrados pelo PMM). Esse número elevaria o quantitativo de médicos em setembro de 2015 para 12.711.

### Despesa Total em Saúde Per Capita

O investimento em saúde, captado pela análise da despesa per capita total em saúde, revela que os municípios com até 10.000 hab., especialmente aqueles com até 5.000 hab., realizaram maiores despesas com saúde por habitante em comparação aos municípios de outros portes populacionais. A Figura 4 ilustra essa realidade, com associação significativa entre as variáveis pelo teste do qui-quadrado (p < 0,05).

**Figura 4:** Número de municípios por porte populacional médio em 2013–2015, segundo quartis da média de despesa total com saúde per capita para o período, em gráfico de colunas 100% empilhadas. Nota: cada quartil representa um conjunto de 224 municípios (25,0% da amostra), em ordem crescente, com o primeiro quartil (Q1) incluindo os municípios com menores despesas per capita e o quarto quartil (Q4), os municípios com maiores despesas per capita.

Fonte: elaboração própria com base nos dados da pesquisa.

A mediana da despesa total na amostra passou de R$ 386,0 no ano de 2013 para R$ 447,7 em 2015 (p < 0,05, Wilcoxon pareado). Em ambos os anos, ela foi maior nos municípios com até 5.000 hab.: R$ 566,1 em 2013 e R$ 661,4 em 2015. Também foi observado que níveis crescentes da produção de consultas médicas pelas eSF se associaram a maiores níveis de investimento per capita em saúde no período de 2013 a 2015 (p < 0,05, qui-quadrado).

## DISCUSSÃO

Os ajustes realizados na preparação do banco de dados mostraram-se adequados, contribuindo para a recuperação de dados sensíveis, que favoreceram a análise. A utilização da escala de saturação de cores do Excel possibilitou identificar inconsistências nos dados informados pelo SIA/SUS, interpretadas como erros ou fragilidades em processos de obtenção, alimentação ou registro de dados no sistema.

A redução ou ápice das consultas médicas na APS em meses cíclicos, observada nos resultados, é tema pouco explorado na literatura, limitando abordagem comparativa. Acredita-se que as reduções estejam relacionadas aos festejos tradicionais (que envolvem o São João no NE, o Natal e o Réveillon), à proximidade de períodos de recesso trabalhista e férias escolares, além do período de consolidação semestral e anual da produção assistencial no SUS.

O aumento das consultas médicas na APS, conforme dados apresentados, corrobora o resultado de estudo multicêntrico realizado pela Rede Observatório do PMM, que analisou dados de 3.000 municípios do país^8^. Entre 2013 e 2015, houve aumento de 33% no número de consultas médicas nos municípios participantes do programa, contra 15% nos municípios não participantes. Neste estudo, o aumento dos municípios participantes foi menor: 19,2%. Como os dados da Rede Observatório foram de abrangência nacional e divulgados de forma agrupada, não foi possível realizar comparações específicas.

Pôde-se verificar o melhor desempenho dos municípios de menor porte populacional na produção de consultas médicas da ESF. Esse desempenho deve refletir não só a maior cobertura da estratégia nesses municípios, mas também a configuração da assistência à saúde. Entende-se que, em municípios de menor porte, a APS tende a ser a principal ou única configuração de rede pública de saúde local, centrada na oferta de assistência médica pela ESF, justificando maior procura por serviços nesse âmbito. Considera-se também a importância do PMM em prover acesso aos municípios com maiores dificuldades em captar profissionais. Nos municípios de maior porte, a maior oferta de médicos fora da ESF, sobretudo de clínicos e pediatras, amplia as opções de porta de entrada e acesso no SUS^9^.

Referentes à produção diária de consultas médicas por profissional, dois pressupostos relacionados à influência do PMM podem esclarecer a redução observada: a maior regularidade da presença dos médicos nos serviços e a diminuição da rotatividade de profissionais. Esses dois fatores tendem a fortalecer o vínculo profissional-usuário, reduzir a insegurança da população quanto à ausência de médicos, minimizar filas e tempo de espera, ordenar a agenda de atendimento e evitar demandas reprimidas^10^. Assim, pelo aumento da confiança da população na oferta regular de assistência médica na ESF, a procura, programada ou espontânea, seria redistribuída nos dias da semana e levaria à redução diária de consultas. Estudo qualitativo realizado em duas comunidades quilombolas referiu que o PMM provocou mudanças. O trabalho destacou a ampliação da frequência dos médicos nos serviços e nas visitas domiciliares, o que repercutiu na otimização dos agendamentos de consulta, facilitando a utilização desses serviços pela população^10^.

Aliado a esses fatores, também se ressaltam como pressupostos a ampliação do tempo de duração das consultas e a melhoria da qualidade da atenção. O acompanhamento supervisionado dos médicos do programa e a inserção de profissionais intercambistas, com formação e experiência de atuação na APS em outros cenários, são elementos passíveis de influenciar mudanças na atuação médica. Não se trata apenas de aumento no número de médicos, mas também de implementação do processo de formação continuada e acompanhamento. Esses elementos necessitam de maior aprofundamento com estudos qualitativos, que poderão auxiliar no aprimoramento do programa.

Em investigação qualitativa realizada com usuários da ESF em Recife (PE), a qualidade da atenção prestada por médicos cubanos foi enfatizada, pela realização de práticas clínicas diferenciadas. Segundo a interpretação dos relatos, o cuidado prestado por eles era caracterizado por “compromisso, escuta qualificada, olhar ampliado sobre o sujeito e valorização dos determinantes sociais do processo saúde-doença”^11^.

Quanto à ampliação da força de trabalho médico na APS a partir do PMM, os resultados deste estudo são corroborados pela literatura. Análise do provimento médico em amostra de 3.755 municípios em todas as regiões do país concluiu que houve maior impacto do PMM em municípios de até 10.000 hab., com aumento de 22,2% da força de trabalho médico^9^. A ampliação significativa da oferta de assistência médica nas eSF nesses municípios foi justificada pela maior demanda, uma vez que ela é organizada predominantemente pela APS.

Sabe-se que, nos municípios de maior porte populacional, pelo maior desenvolvimento socioeconômico e concentração de serviços de média e alta complexidades, há mais atrativos para a fixação de médicos^12^. Nesse sentido, esses municípios dependem menos de políticas de provimento, fato que justifica a maior proporção de médicos do PMM nas eSF dos municípios de menor porte populacional^13^. Acerca do aumento real do quantitativo de médicos nas eSF ter sido menor do que o esperado, outros trabalhos também relataram essa situação, justificando substituição de profissionais ou reposição de médicos em equipes incompletas^9 , 13 , 14 , 15^.

O aumento da cobertura de eSF em municípios de todos os portes populacionais, com aproximação de 100% naqueles com até 20.000 habitantes, revela a importância do PMM para suprir a demanda por assistência médica na APS, tendo influência direta na ampliação das consultas no período observado. Esse resultado converge com os dados apresentados em estudo de amplitude nacional, que revelou aumento de 16,2% na cobertura das eSF de 2012 para 2015^15^. As maiores coberturas foram observadas nos municípios com menos de 30.000 hab., com média de 98,4% em 2015; nos municípios entre 100.000 e 200.000 hab., a média correspondeu a 48,9%.

No tocante à análise do investimento em saúde, destaca-se o maior investimento per capita dos municípios de menor porte populacional. Apesar do PMM possibilitar a desoneração dos municípios em relação aos gastos com provisão de médicos^16^, o aumento nesses municípios tende a ser reflexo da maior produção de consultas médicas na ESF e da ampliação da cobertura de eSF.

Por se tratar de investigação consolidada a partir do uso de dados secundários, com registros de consultas médicas em escala regional, reconhecem-se tais aspectos como limitações do método neste estudo. Desse modo, recomenda-se a realização de pesquisa que possa explorar o contexto local da APS e as possíveis repercussões nas ações e serviços considerando as diretrizes atuais.

## CONCLUSÕES

Os resultados deste estudo mostram evidências de que houve aumento da produção de consultas médicas na APS no NE, caracterizando ampliação do acesso e da utilização de serviços nesse âmbito a partir da implementação do PMM. Entre abril de 2013 e setembro de 2015, houve efetivo aumento da cobertura de eSF, constituindo importante conquista para o SUS.

O melhor desempenho na produção de consultas médicas obtido pelos municípios de menor porte populacional, em função de maiores investimentos per capita com saúde e da centralidade da APS na oferta de ações e serviços, sugere a necessidade de se avançar na discussão da alocação equitativa de recursos na saúde e de se fortalecer as redes de atenção, com ênfase na regionalização.

Os achados podem contribuir com as recentes discussões acerca dos efeitos do PMM, à medida que evidencia a influência do programa em diferentes contextos. As futuras alternativas ao programa a serem implantadas podem, a partir dos avanços e dificuldades do PMM, investir em estratégias que aumentem os resultados positivos alcançados e minimizem os limites, a fim de dar continuidade ao acesso da população na APS.
